# MOCDT: multi-cancer detection and tissue-of-origin classification via cfDNA multi-modal integration

**DOI:** 10.1093/bioinformatics/btag274

**Published:** 2026-07-07

**Authors:** Gihyeon Kim, Seungyeon Rhee, Yumi Lee, Seongmun Jeong, Tae-You Kim, Jang-Hwan Choi

**Affiliations:** Department of Artificial Intelligence, Ewha Womans University, Seoul 03760, Republic of Korea; Division of Artificial Intelligence & Software, Ewha Womans University, Seoul 03760, Republic of Korea; Division of Artificial Intelligence & Software, Ewha Womans University, Seoul 03760, Republic of Korea; IMBdx Inc., Seoul 08506, Republic of Korea; IMBdx Inc., Seoul 08506, Republic of Korea; Cancer Research Institute, Seoul National University, Seoul 03080, Republic of Korea; Department of Artificial Intelligence, Ewha Womans University, Seoul 03760, Republic of Korea; Division of Artificial Intelligence & Software, Ewha Womans University, Seoul 03760, Republic of Korea

## Abstract

**Motivation:**

Tumor-derived circulating tumor DNA (ctDNA) fragments present in blood provide rich molecular signals for identifying cancer and mapping its tissue of origin. However, leveraging these heterogeneous signals requires robust computational integration methods. Existing multi-modal approaches often fail to capture both inter-modality structure and inter-patient relationships, limiting their utility for robust cancer detection (CD) and fine-grained tissue-of-origin (TOO) classification.

**Results:**

We propose MOCDT, a cell-free DNA (cfDNA) multi-omics framework that follows a clinically aligned two-stage pipeline: high-specificity CD followed by conditional TOO classification. MOCDT combines (i) a supervised multi-modal autoencoder incorporating adversarial modality alignment and supervised contrastive geometry shaping, with (ii) a latent space patient similarity network and (iii) a residual Graph Convolutional Network for relational learning. Applied to a cfDNA cohort including healthy controls and eight cancer types, MOCDT achieved 95.74% specificity and 96.22% sensitivity for CD at a high-specificity operating point, and 75.2% Top1 and 91.06% Top3 accuracy for TOO classification. Latent attribution analysis showed that the model learns tissue-dependent latent features rather than relying on a single universal biomarker axis. Together, these results demonstrate that MOCDT enables accurate and interpretable cfDNA-based multi-omics integration, supporting clinically relevant liquid biopsy applications.

**Availability and implementation:**

Code and Dataset are available at https://github.com/Ewha-AI/MOCDT.

## 1 Introduction

Blood-based liquid biopsy has attracted considerable attention as a non-invasive approach for early cancer detection (CD) and tissue-of-origin (TOO) prediction (Liu *et al.* 2020, Lo *et al.* 2021). The need for multi-cancer early detection (MCED) and multi-modal approaches stems from the molecular diversity of tumors, which share common oncogenic pathways yet exhibit distinct epigenetic landscapes shaped by their cellular origins. In particular, circulating tumor DNA (ctDNA) within cell-free DNA (cfDNA) captures tumor-derived genomic and epigenomic alterations, providing cues for both CD and TOO inference.

cfDNA methylation patterns capture epigenomic alterations carried by ctDNA and remain relatively stable from early stages of tumorigenesis, making them well-suited for early CD (Shen *et al.* 2018, Cristiano *et al.* 2019). In contrast, copy number variation (CNV) directly reflects structural genomic instability and can be reliably reconstructed at the genome-wide level even from low-depth cfDNA sequencing, which is especially beneficial when ctDNA abundance is low (Van Roy *et al.* 2017, Chen *et al.* 2019). More recently, fragmentomics (Cristiano *et al.* 2019), particularly cfDNA fragment length distributions and fragment size ratios (FSR), has emerged as a complementary diagnostic signal (Tsui *et al.* 2025). Tumor-derived cfDNA tends to exhibit an overrepresentation of shorter fragments due to aberrant cell death processes, and this property is largely independent of genomic sequence information. Although methylation, CNV, and FSR are all derived from cfDNA, each captures a distinct aspect of the ctDNA signal. Single-modality approaches may fail to reflect the full spectrum of malignant transformation, especially in early-stage tumors with low ctDNA abundance. In contrast, multi-modal frameworks integrate heterogeneous biological signals to provide a more comprehensive representation of tumor biology (Wang *et al.* 2021, Li *et al.* 2022, Dou and Mirzaei 2025). Recent studies have shown that deep learning–based multi-modal integration can improve performance in CD and TOO prediction (Liu *et al.* 2024).

### 1.1 Multi-omics integration via representation and relation learning

Recent studies have pointed out that unsupervised multi-omics integration methods (Valpola 2015, Hira and Gillies 2015) fail to adequately capture the complex inter-omics correlations (Dou and Mirzaei 2025). While these approaches can effectively project different omics into a shared representation space, they often struggle to distinguish predictive molecular signals from background variation, leading to limited performance gains in downstream tasks. To address this limitation, semi-supervised approaches that combine unsupervised representation learning with supervised objectives have been proposed. For example, MOANNA (Lupat *et al.* 2023) integrates an autoencoder-based latent representation with a classification loss and reports significant improvements in molecular subtype classification compared with single-omics models.

Meanwhile, conventional feature-level integration methods suffer from another limitation in that they treat patients as independent samples and therefore do not explicitly exploit inter-patient relationships. To overcome this issue, Similarity Network Fusion (SNF) (Wang *et al.* 2014) was introduced to integrate patient-wise similarities computed from each omics modality into a unified network, where patients are represented as nodes and pairwise similarities as edges, providing a graph-based relational prior. Building on this idea, MOGONET (Wang *et al.* 2021) and MoGCN (Li *et al.* 2022) proposed frameworks that learn SNF-constructed patient similarity graphs using Graph Convolutional Networks (GCNs), demonstrating that explicitly modeling inter-patient relationships leads to improved classification performance in multi-omics analysis. Collectively, these studies highlight the importance of jointly considering representation learning and relational modeling in multi-omics integration.

### 1.2 Representation alignment for robust multi-omics integration

In heterogeneous multi-modal and multi-omics settings, differences in measurement characteristics, such as dynamic range, noise profiles, and missingness patterns, can induce a modality gap that biases fused representations toward dominant or cleaner modalities (Zitnik *et al.* 2019). This bias undermines the stability of multi-modal integration and degrades generalization under distribution shifts. To mitigate this issue, adversarial representation alignment encourages modality-invariant embeddings by training encoders to confuse a modality discriminator (Ganin *et al.* 2016), thereby reducing modality-induced distributional discrepancies in a shared latent space (Wu *et al.* 2023, Yinghua *et al.* 2025).

Complementary to modality alignment, contrastive learning aims to explicitly shape the latent geometry to be more favorable for downstream discrimination. In high-dimensional, small-cohort regimes, standard classification losses alone may not sufficiently enforce class separation, resulting in partially overlapping latent clusters and ambiguous decision boundaries—particularly for fine-grained TOO classification. Supervised contrastive formulations based on triplet objectives directly impose relative distance constraints among anchor–positive–negative samples, promoting intra-class compactness and inter-class separation (Bellet *et al*. 2013, Khosla *et al.* 2020). Such formulations have been reported to improve robustness and class separability in cancer-related liquid biopsy and multi-omics prediction settings (Sharifi-Noghabi *et al.* 2019, Park *et al*. 2021, Karimzadeh *et al.* 2024).

Against this background, we present MOCDT, a cfDNA-based multi-omics framework for multi-CD and TOO classification. MOCDT adopts a unified representation learning paradigm tailored to heterogeneous cfDNA signals, followed by graph-based learning to support robust normal–cancer prediction and conditional TOO classification. The main contributions of this work are summarized as follows:

- We propose MOCDT, a cfDNA-based multi-omics framework with a clinically aligned two-stage pipeline supporting high-specificity CD followed by conditional TOO classification.
- We introduce a multi-modal representation learning strategy that combines adversarial domain confusion and triplet-based contrastive alignment within a supervised autoencoder, yielding modality-robust and class-discriminative latent representations.
- We construct a latent space patient similarity network and perform graph-based prediction with a lightweight residual GCN, leveraging cohort-level relational structure while reducing sensitivity to ultra-high-dimensional input noise.

## 2 Materials and methods

### 2.1 Data preparation

#### 2.1.1 Study design and sample collection

This study employed a retrospective cohort of patients diagnosed with eight cancer types, including colorectal, gastric, liver, pancreatic, lung, breast, ovarian, and prostate, along with a cohort of healthy individuals. The study dataset was assembled from seven participating institutions, with detailed cohort characteristics summarized in Table 1, available as supplementary data at *Bioinformatics* online. Participants were recruited following approval by the Institutional Review Boards of the participating centers. Eligibility for cancer patients required histological confirmation of the disease type and stage according to the 8th edition of the American Joint Committee on Cancer guidelines. The healthy control group consisted of individuals with no prior history of malignancy or major chronic diseases. Plasma samples from cancer patients were collected prior to the initiation of any treatment, while whole blood samples were obtained from healthy subjects.

**Table 1:** Performance comparison of CD and TOO classification with benchmark models.^a^

	Cancer detection			TOO (GT cancer)	TOO (predicted cancer)			# of predicted
	Accuracy	Specificity	Sensitivity	Accuracy	Top1 Acc	Top2 Acc	Top3 Acc	Cancer
MoGCN	0.6657	**0.9681**	0.5527	0.2803	0.3237	0.5396	0.6691	284
MOGONET	0.5790	0.9260	0.4490	0.3976	0.4167	0.5625	0.6667	240
MO-GCAN	0.6151	0.6915	0.5865	0.3042	0.2720	0.4164	0.4901	353
MOCDT (Proposed)	**0.9609**	0.9574	**0.9622**	**0.7645**	**0.7520**	**0.8679**	**0.9106**	492

a For the TOO evaluation, Top-*K* accuracy measures whether the ground-truth cancer type is contained within the top-*K* predicted classes ranked by posterior probability, reflecting clinically relevant diagnosis scenarios. CD thresholds were calibrated on validation folds to match the target specificity; reported specificity/sensitivity are measured on the test set. Bold values indicate the best performance for each metric.

#### 2.1.2 Sample processing and cfDNA extraction

Plasma was separated from whole blood using standard two-step centrifugation. cfDNA was extracted using the Maxwell^®^ RSC ccfDNA Plasma Kit (Promega) following the manufacturer’s protocol, and DNA quantity and integrity were assessed using an Agilent 4200 TapeStation system. Genomic DNA from tumor and matched normal tissues was extracted using the Maxwell^®^ RSC Tissue DNA Kit. Detailed sample processing and quality control procedures are provided in the Supplementary Material, available as supplementary data at *Bioinformatics* online.

#### 2.1.3 Library preparation and sequencing

Methylation-sequencing libraries were constructed using the IMBdx AlphaLiquid^®^ screening platform, which employs enzymatic conversion to preserve DNA integrity. Libraries were sequenced on the Illumina NovaSeq 6000 platform. This process yielded approximately 100 Gb of raw data per sample with 150 bp paired-end reads.

#### 2.1.4 NGS data preprocessing

cfDNA bisulfite sequencing reads were preprocessed using standard pipelines (QC, trimming, alignment to GRCh38, and deduplication). Detailed parameters, software versions, and quality control criteria are provided in the Supplementary Material, available as supplementary data at *Bioinformatics* online.

#### 2.1.5 DNA methylation analysis

To quantify DNA methylation levels, beta values for all CpG sites were calculated using MethylDackel (v0.6.1). To ensure the stability of the methylation calls and minimize stochastic errors in low-depth regions, a minimum read depth filter of 3 was applied; CpG sites with coverage below this threshold were excluded from the analysis. To facilitate comparative analysis with established epigenetic benchmarks, we selectively extracted beta values for CpG sites corresponding to the coordinates of the Illumina Infinium HumanMethylation450 (450K) BeadChip. This integration allowed for a robust assessment of methylation patterns across biologically significant and well-annotated genomic regions.

#### 2.1.6 Characterization of copy number patterns

To identify copy number alterations, copy number ratios (CNRs) were calculated using the R package “QDNAseq” over non-overlapping bins. Reads within each bin were counted, and bins overlapping with the GRCh38 blacklist regions were excluded. GC-content bias and mappability were corrected, followed by normalization and smoothing to generate the CNRs. Bin segmentation and calling of CNVs were performed using default parameters.

#### 2.1.7 Fragment size ratio

FSR was analyzed as a key fragmentomic feature to differentiate tumor-derived cfDNA from background DNA. cfDNA fragments were categorized into short (>80 and <150 bp) and long (>150 and <220 bp) intervals. The FSR was defined as the ratio of short to long fragments, leveraging the biological observation that tumor-derived fragments tend to be shorter due to aberrant apoptotic cleavage. Fragment length extraction and binning were performed using SAMtools. The ratios were standardized across samples to account for variations in total cfDNA yield and were subsequently integrated into an ensemble machine learning framework to enhance CD sensitivity.

### 2.2 Data preprocessing

We processed multi-omics cfDNA data, including DNA methylation, CNV, and FSR obtained from healthy individuals and patients with cancer. The cohort consisted of 1423 samples, split into train (*n* = 732) and test (*n* = 691) sets with balanced class ratios. An overview of the class distribution for the train and test sets is provided in Table 2, available as supplementary data at *Bioinformatics* online.

**Table 2:** Performance comparison of ablation experiments.^a^

	Cancer detection			TOO (GT cancer)	TOO (predicted cancer)
	**Accuracy**	**Specificity**	**Sensitivity**	**Accuracy**	**Accuracy**
MOCDT (Proposed)	0.9609	**0.9574**	0.9622	**0.7645**	**0.7520**
(-)ResGCN	0.9682	**0.9574**	0.9722	0.7587	0.7465
(-)ResGCN (-)Latent SNF	0.9667	**0.9574**	0.9702	0.7582	0.7460
(-)ResGCN (-)Latent SNF (-)Cont	**0.9696**	**0.9574**	0.9742	0.7388	0.7269
(-)ResGCN (-)Latent SNF (-)Cont (-)Adv	**0.9696**	0.9362	**0.9821**	0.7247	0.7075

a (-)Adv, no adversarial learning; (-)Cont, no contrastive learning; (-)ResGCN, no residual layers for GCN; (-)Latent SNF, use raw input data to construct SNF. Bold values indicate the best performance for each metric.

To address missing values, modality-specific preprocessing procedures were applied. For DNA methylation, missing beta values were imputed using MethylGPT (Ying *et al.* 2024), a transformer-based foundation model pretrained on over 150 000 human methylation profiles. We constructed a methylation feature set by combining CpG sites used in MethylGPT pretraining with those without missing values, resulting in 363 987 CpGs. From this set, supervised feature selection was performed using limma (Ritchie *et al.* 2015) with a binary cancer label (*P*<.05), yielding 110 392 CpG features. For FSR, regions with more than 50% missing values were removed, retaining 25 461 features, while all 24 579 CNV features were used. To prevent information leakage, the feature set was defined using the train set only, and the test set was restricted to the selected feature subset. For methylation, the original methylation signals were used during model training, with missing selected values filled with zero. For FSR, all missing values were also replaced with zero. Detailed preprocessing procedures are provided in the Supplementary material, available as supplementary data at *Bioinformatics* online.

### 2.3 Overview of MOCDT framework

**Figure 1:**
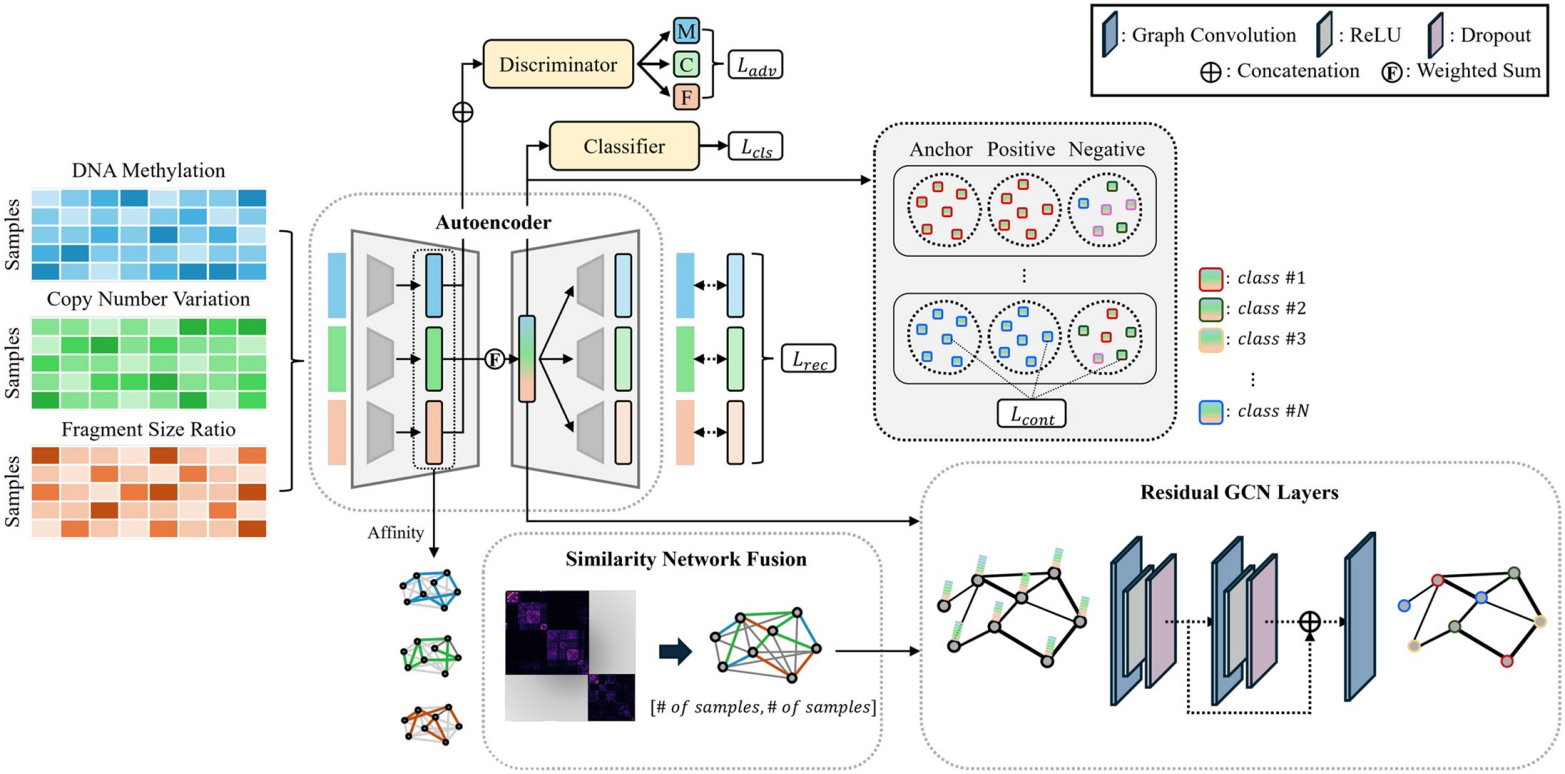
The overall framework of MOCDT. The model consists of a supervised multi-modal autoencoder, a fused SNF network based on latent representations, and GCN layers. The GCN utilizes the fused latent and fused similarity network matrix to make a final prediction on CD and TOO classification. C, copy number variation; F, fragment size ratio; $L_{\text{adv}}$, adversarial loss; $L_{\text{cls}}$, classification loss; $L_{\text{rec}}$, reconstruction loss; $L_{\text{cont}}$, contrastive triplet loss; M, DNA methylation.

The proposed MOCDT framework consists of three main components: (i) a supervised multi-modal autoencoder, (ii) SNF applied in the latent space, and (iii) residual GCN layers. First, a supervised multi-modal autoencoder takes three cfDNA modalities as input and learns both modality-specific latent representations and a fused latent embedding. Next, SNF is performed on the fused latent features to construct a patient similarity network (PSN). Finally, a GCN module jointly utilizes the fused latent features and the PSN to make the final prediction. The inference procedure of MOCDT follows a clinically realistic workflow: the model first performs CD and subsequently predicts the TOO for patients predicted as cancer-positive. This two-step design reflects the clinical utility of cfDNA-based multi-cancer early detection assays, where CD and TOO classification are sequential decision stages. The overall architecture of the MOCDT framework is illustrated in Fig. 1.

### 2.4 Supervised multi-modal autoencoder

To address the limited sample size and high-dimensional input space inherent in cfDNA-based multi-omics data, we designed a supervised multi-modal autoencoder to learn compact latent representations across three modalities. For each modality, an individual encoder composed of a single hidden layer with 750 units, batch normalization, and a sigmoid activation function was used to project the input features into a latent space. The modality-specific latent vectors were then fused using fixed weights of 2:4:4 for methylation, CNV, and FSR, respectively. Each modality was reconstructed from the fused latent representation via a single-layer decoder. The latent dimension and fusion weights were determined through hyperparameter tuning, with the final ratio selected via grid search.

To incorporate supervised information, a classifier composed of three hidden layers (128, 32 units with dropout of 0.3) was applied to the fused latent representation. The classifier was trained using nine-class labels (healthy and eight cancer types), encouraging the fused latent embeddings to form disease-specific clusters rather than merely optimizing reconstruction quality (Fig. 1, available as supplementary data at *Bioinformatics* online). The total loss function consists of four components:


(1) $$L_{\text{total}}=L_{\text{rec}}+L_{\text{cls}}-\lambda_{\text{adv}}L_{\text{adv}}+\lambda_{\text{con}}L_{\text{cont}},$$

where

-
$L_{\text{rec}}$ is the reconstruction loss,
-
$L_{\text{cls}}$ is the supervised classification loss,
-
$L_{\text{adv}}$ is the adversarial domain confusion loss, and
-
$L_{\text{cont}}$ is the contrastive triplet loss.

Reconstruction was optimized via mean squared error between the original and reconstructed feature matrices. This encourages the model to preserve global cfDNA signal structures beyond purely cancer-associated patterns, functioning as a form of self-regularization that mitigates overfitting and improves generalization under small-sample conditions.

#### 2.4.1 Adversarial domain confusion

To learn modality-invariant representations, adversarial learning was introduced on the modality-specific latent vectors. For each batch, the three modality-specific latent embeddings were concatenated, and the discriminator was trained to predict the modality label using a cross-entropy loss function. The encoder then maximized $L_{\text{adv}}$ to fool the discriminator, thereby inducing domain confusion and reducing modality-induced distributional shifts. This mechanism preserves modality-specific biology while promoting a shared latent space that stabilizes downstream tasks.

#### 2.4.2 Contrastive feature alignment

To enhance class-level separation, triplet contrastive learning was applied to the fused latent embeddings using the nine-class labels. Anchor–positive–negative triplets were sampled to minimize intra-class distances and maximize inter-class distances. This improves discrimination between healthy and cancer latent embeddings and mitigates class overlap among different cancer types, contributing to improved CD sensitivity at high-specificity regions and reducing TOO misclassification.

#### 2.4.3 Training configuration

Two Adam optimizers were used during training, one for updating the encoder–decoder–classifier parameters and another for updating the discriminator. In each iteration, the discriminator was updated first, followed by the encoder–decoder–classifier update. Models were trained for 100 epochs with a batch size of 32 and a learning rate of $1\times 10^{-3}$. The train set was randomly split into train and validation subsets, and the final model was selected based on minimum validation loss. The best-performing configuration was obtained with $\lambda_{\text{adv}}$=0.05 and $\lambda_{\text{con}}$=0.2, which enabled cross-modality robustness while preserving cancer-relevant modality-specific patterns.

### 2.5 Latent space patient similarity network

In this study, we construct modality-specific sample similarity graphs from the latent representations learned by a multi-modal autoencoder and integrate them via SNF to obtain a single fused graph. Given a trained autoencoder, we extract modality-wise latent vectors for each sample and organize them into modality-specific latent representation matrices for each modality m∈{1,…,M}.

#### 2.5.1 Train-based latent preprocessing

To ensure stable and comparable affinity estimation across modalities, we apply a series of train-based preprocessing steps to the latent representations. For each modality, a StandardScaler is fit on the train samples and applied to both train and test splits to enforce consistent scaling. To further reduce the influence of extreme values and degenerate dimensions, we perform range clipping and remove near-constant latent features, identified as dimensions with very small standard deviations on the train set. All preprocessing transformations and feature masks are derived exclusively from the train set and applied identically across splits, thereby preserving a consistent latent feature space and preventing information leakage.

#### 2.5.2 Modality-specific affinity graph construction

Using the preprocessed modality-wise latent representations, we compute modality-specific sample–sample affinity matrices ${\left\{W^{\left(m\right)}\right\}}_{m=1}^{M}$. Pairwise affinities are computed using a Gaussian kernel over squared Euclidean distances and sparsified by retaining only the *K*-nearest neighbors (K=15).


(2) $$W_{ij}^{\left(m\right)}=\text{exp}\;\left(-\frac{\|x_{i}^{\left(m\right)}-x_{j}^{\left(m\right)}{\|}_{2}^{2}}{\sigma_{m}^{2}}\right).$$

In addition, kernel scaling parameters are applied to normalize the affinity scale across modalities.

#### 2.5.3 Similarity network fusion

SNF (Wang *et al.* 2014) is a widely adopted framework for integrating patient-level similarity networks derived from multiple data modalities. In SNF, information is iteratively exchanged across the modality-specific affinity networks ${\left\{W^{\left(m\right)}\right\}}_{m=1}^{M}$, yielding a single integrated network $W^{\text{fused}}$. This step does not involve supervised learning or parameter optimization; rather, it deterministically computes an integrated sample similarity matrix from the given affinity matrices. The fused matrix provides, for each sample pair (i,j), a consensus weight $W_{ij}^{\text{fused}}$ that reflects cross-modality agreement.

Finally, $W^{\text{fused}}$ is organized as a matrix indexed by sample ids, and split-specific submatrices are extracted according to the train and test sample lists and saved as adjacency matrices for downstream modeling. To limit the effect of self-connections, diagonal entries are set to zero. The resulting fused graph is subsequently used as the adjacency input to the residual GCN.

### 2.6 Residual graph convolutional network for classification

We employ a residual GCN classifier to implement a clinically aligned decision flow from multi-modal cfDNA inputs by modeling each sample as a node and leveraging cohort-level structure via a sample–sample similarity graph. Graph-based learning stabilizes predictions under sparse and noisy cfDNA signals by propagating information among similar samples, while residual connections mitigate representation degradation and optimization instability during repeated graph propagation.

#### 2.6.1 Graph construction and node features

The node feature matrix *X* is defined by the fused latent representation obtained from the supervised multi-modal autoencoder and SNF-based integration. In parallel, modality-specific affinities computed in latent spaces [Equation (2)] are integrated via SNF to produce a single similarity matrix, from which a sparse adjacency matrix *A* is constructed. The residual GCN thus takes both fused sample representations and the inter-sample graph structure as an input.

#### 2.6.2 Residual GCN architecture

To stabilize graph propagation, we adopt the standard GCN formulation with self-loops and symmetric degree normalization. Given an adjacency matrix *A*, we define $\tilde{A}=A+I$ and ${\tilde{D}}_{ii}=\sum_{j}{\tilde{A}}_{ij}$, and use the normalized adjacency


$$\hat{A}={\tilde{D}}^{-\frac{1}{2}}\tilde{A}{\tilde{D}}^{-\frac{1}{2}}.$$

A graph convolution operation is defined as


$$\text{GC}(H,\hat{A})=\hat{A}HW+b,$$

where *W* and *b* are learnable parameters.

The residual GCN comprises three graph convolution layers. ReLU activation and dropout (0.1) are applied after the first two layers, with a residual connection at the second layer:


(3) $$\begin{array}{c} H_{1}=\text{Dropout}(\text{ReLU}({\text{GC}}_{1}(X,\hat{A}))),\\ {\tilde{H}}_{2}=\text{Dropout}(\text{ReLU}({\text{GC}}_{2}(H_{1},\hat{A}))),\\ H_{2}={\tilde{H}}_{2}+H_{1},\;Z={\text{GC}}_{3}(H_{2},\hat{A}). \end{array}$$

Here, *Z* denotes the output logits, with dimensionality N×2 for CD and N×8 for TOO.

#### 2.6.3 Two-stage CD–TOO protocol

Following the clinical decision flow, CD and TOO classification are performed sequentially.

1. CD: Threshold-calibrated binary prediction

The CD model outputs the cancer probability $P_{\text{cancer}}$. To control false positives, we first calibrate a decision threshold to satisfy a predefined target specificity. Specifically, we conduct stratified 4-fold cross-validation within the train set. For each fold, a binary residual GCN is initialized and trained, and the validation probabilities are collected. Aggregating scores across folds, we select a single threshold τ such that the validation specificity meets the target value. After threshold calibration, the final CD model is trained using the full train set under the fixed decision threshold τ. At inference, samples are classified as cancer-positive if their predicted cancer probability exceeds the calibrated threshold τ, i.e., ${\hat{y}}_{\text{CD}}=I[P_{\text{cancer}}\geq \tau ]$, where I[·] denotes the indicator function. The threshold τ was selected on validation folds to meet a target specificity of 0.99; all reported metrics are computed on the held-out test set.

2) TOO: cancer-only multi-class classification

TOO prediction is formulated as a cancer-only multi-class classification task, focusing model capacity on discriminating among tumor origins. The TOO classifier is trained exclusively on ground-truth cancer samples using the standard multi-class cross-entropy loss over the eight TOO classes.

At inference, TOO prediction is applied conditionally following the CD stage. For evaluation, we report performance on both all ground-truth cancer samples and the subset predicted as cancer by CD, capturing intrinsic origin discrimination and end-to-end pipeline performance.

## 3 Experiments and results

### 3.1 Evaluating MOCDT across various cancer types

**Figure 2:**
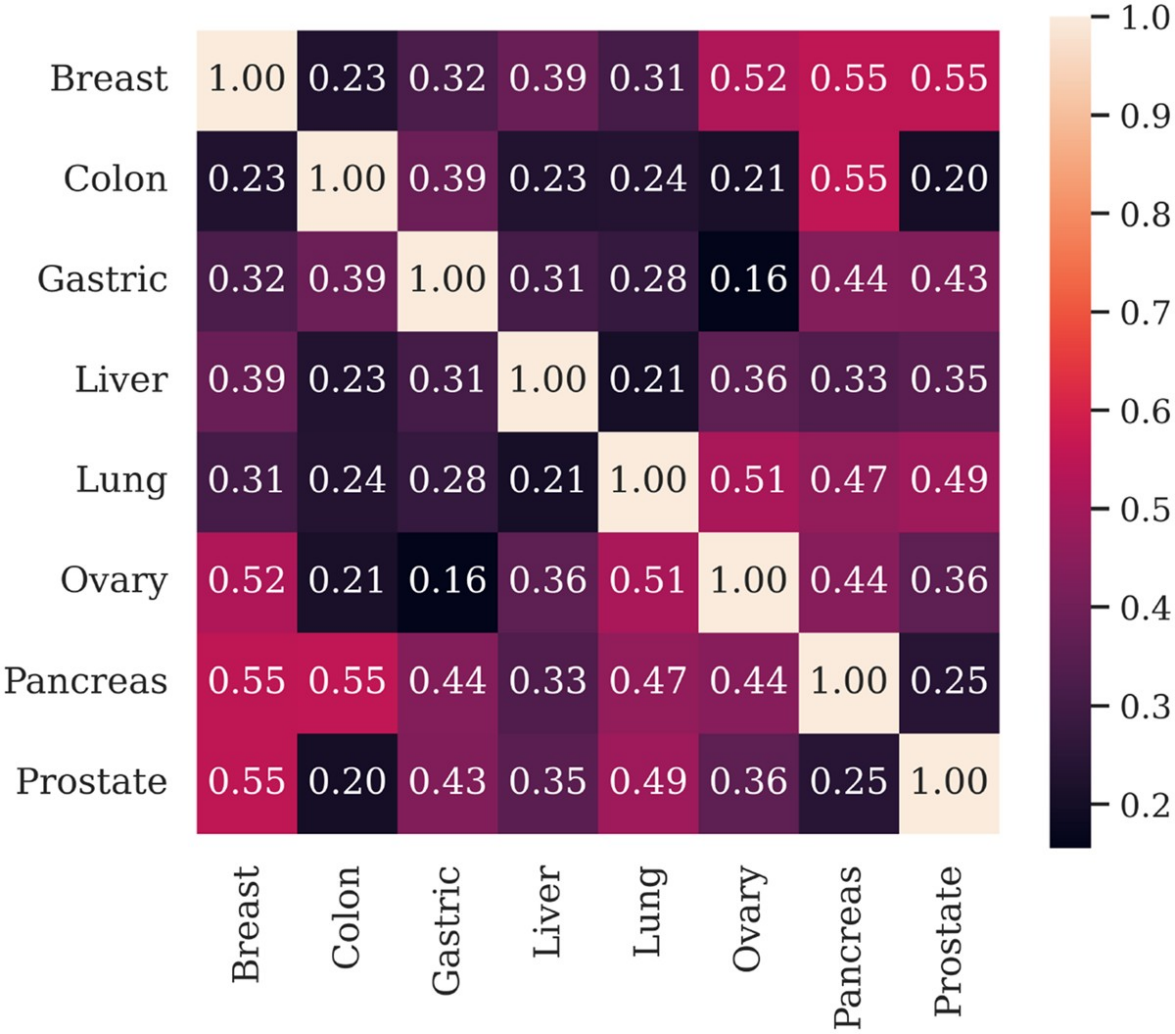
Pearson correlation matrix of mean feature importance profiles across eight cancer types, revealing structured similarity patterns and tissue-specific differences.

MOCDT achieved the strongest overall CD performance (Acc 0.9609, Spec 0.9574, Sens 0.9622) and produced 492 cancer-positive predictions, closely matching the ground-truth cancer count (503), as summarized in Fig. 2, available as supplementary data at *Bioinformatics* online, and Table 1.

Further analysis of TOO prediction stratified by gender (Table 4, available as supplementary data at *Bioinformatics* online) showed consistently stable performance across female and male patients. Female patients achieved an average precision and recall of 0.7183 and 0.7136, respectively, while male patients achieved 0.7986 and 0.7873. These results indicate that MOCDT does not rely on sex-specific biases and provides robust TOO classifications across sexes. Notably, for breast cancer in female patients, MOCDT attained a high precision of 0.9143, reflecting a low false-positive rate and highlighting its potential to reduce unnecessary diagnostic procedures while supporting targeted clinical decision-making.

Stage-stratified analysis of TOO performance (Table 5, available as supplementary data at *Bioinformatics* online) further revealed a recall of 0.8130 in early-stage disease and 0.6894 in late-stage disease. The strong recall observed in early-stage patients is particularly meaningful in the context of liquid biopsy, as it demonstrates MOCDT’s ability to detect TOO signals under low ctDNA abundance. Together, these findings suggest that MOCDT captures subtle tissue-specific molecular patterns beyond tumor burden alone, supporting its applicability to early CD and clinically reliable TOO inference.

### 3.2 Interpretation of latent space

To interpret which cfDNA features drive the binary CD model, we computed node-wise feature attributions based on gradient-based feature attribution adapted to our GCN. Let $f(X,A)\in R^{N\times 2}$ denote the trained GCN producing logits $z_{i}\in R^{2}$ for each node *i*. For a node *i* and a target class c∈{0 (normal), 1 (cancer)}, we first selected the corresponding scalar logit $z_{i,c}$ and computed the gradient of this logit with respect to the input feature vector $x_{i}\in R^{F}$. The importance of feature *f* for node *i* and class *c* was then defined as the absolute input×gradient magnitude:


(4) $$I_{i,c,f}=\left|x_{i,f}\cdot \frac{\partial z_{i,c}}{\partial x_{i,f}}\right|$$

Attributions were averaged within each predicted class over the test set. The resulting class-specific importance scores provide a global ranking of latent features that most strongly contribute to the model’s normal and cancer predictions, respectively.

**Figure 3:**
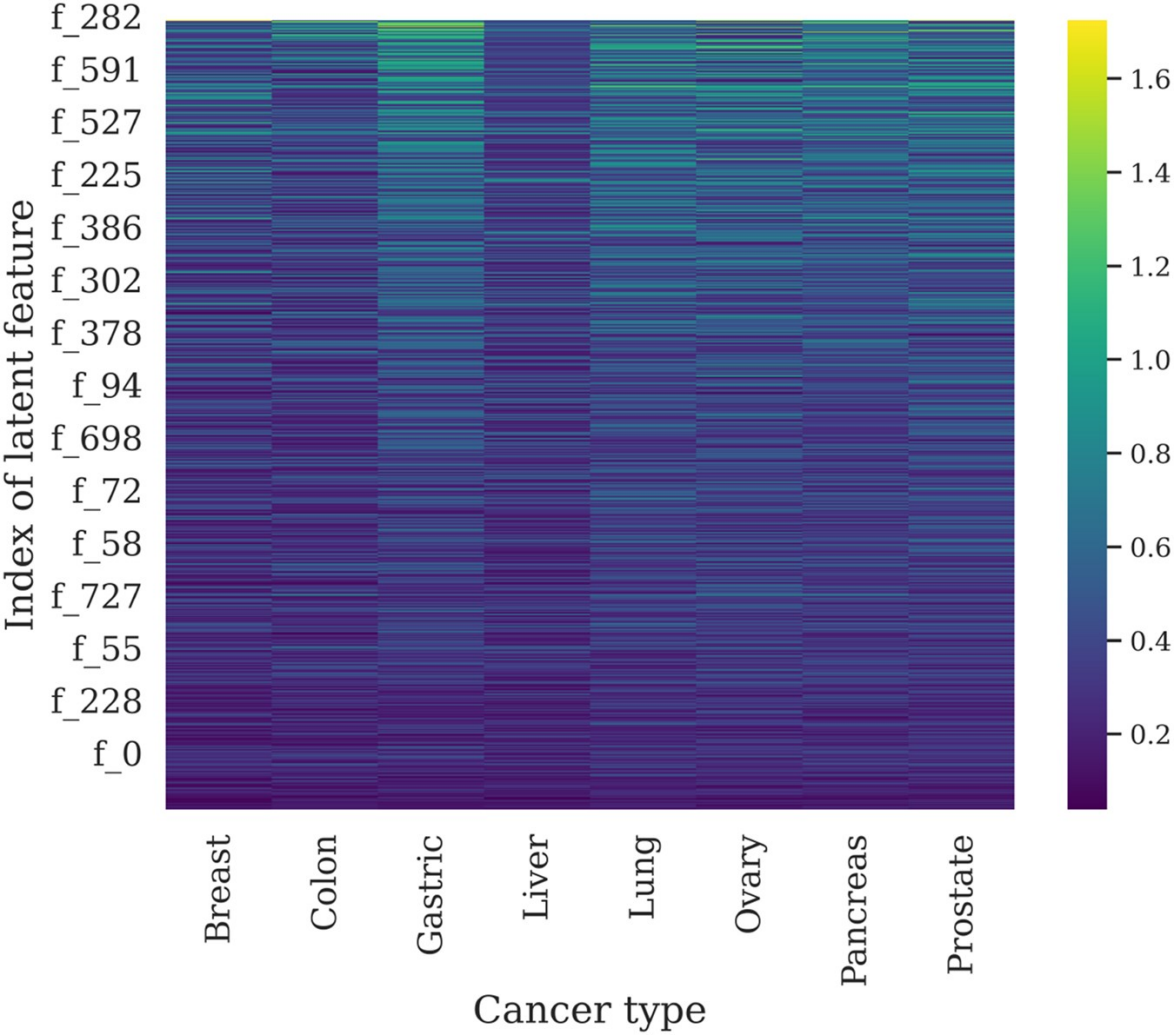
Heatmap of mean feature importance sorted by cross-cancer variance. f_# denotes the index of each latent feature.

When comparing mean latent feature importance between non-cancer and cancer samples (Fig. 3, available as supplementary data at *Bioinformatics* online), most latent dimensions exhibited higher importance for non-cancer, lying below the y=x diagonal. These results indicate that the binary GCN primarily relies on latent axes encoding baseline cfDNA patterns of healthy individuals, treating cancer samples as deviations from this normal structure rather than via distinct cancer-specific axes. This behavior is consistent with reconstruction-based representation learning, in which healthy cfDNA forms a compact latent manifold, whereas cancer cfDNA exhibits greater heterogeneity.

To interpret the TOO, we restricted the analysis to test samples that were predicted as cancer by the binary model and had a ground-truth cancer label. The mean feature importance for each cancer type was computed in the same manner as in the binary setting. To capture the similarity of importance profiles across cancer types, we calculated Pearson correlation coefficients between cancer types and observed distinct correlation patterns (Fig. 2). For instance, breast, ovary, and prostate cancers exhibited moderate correlation (r≈0.36–0.55), suggesting that shared cfDNA fragmentomics features or generally low-shedding cfDNA patterns can be exploited by the model for TOO classification (Lu *et al*. 2025). Colon, gastric, and liver cancers showed partially overlapping profiles (r≈0.23–0.39), consistent with their gastrointestinal origin and previously reported similarities in cfDNA methylation patterns (Lei *et al.* 2024). Lung cancer displayed an intermediate correlation with multiple cancer types (r≈0.21–0.51), which is consistent with heterogeneous cfDNA shedding patterns in lung cancer liquid biopsy studies (Makar *et al.* 2025). Notably, ovary and gastric cancers exhibited very low similarity (r=0.16), indicating highly distinct tissue-specific cfDNA signals captured in the latent space.

**Figure 4:**
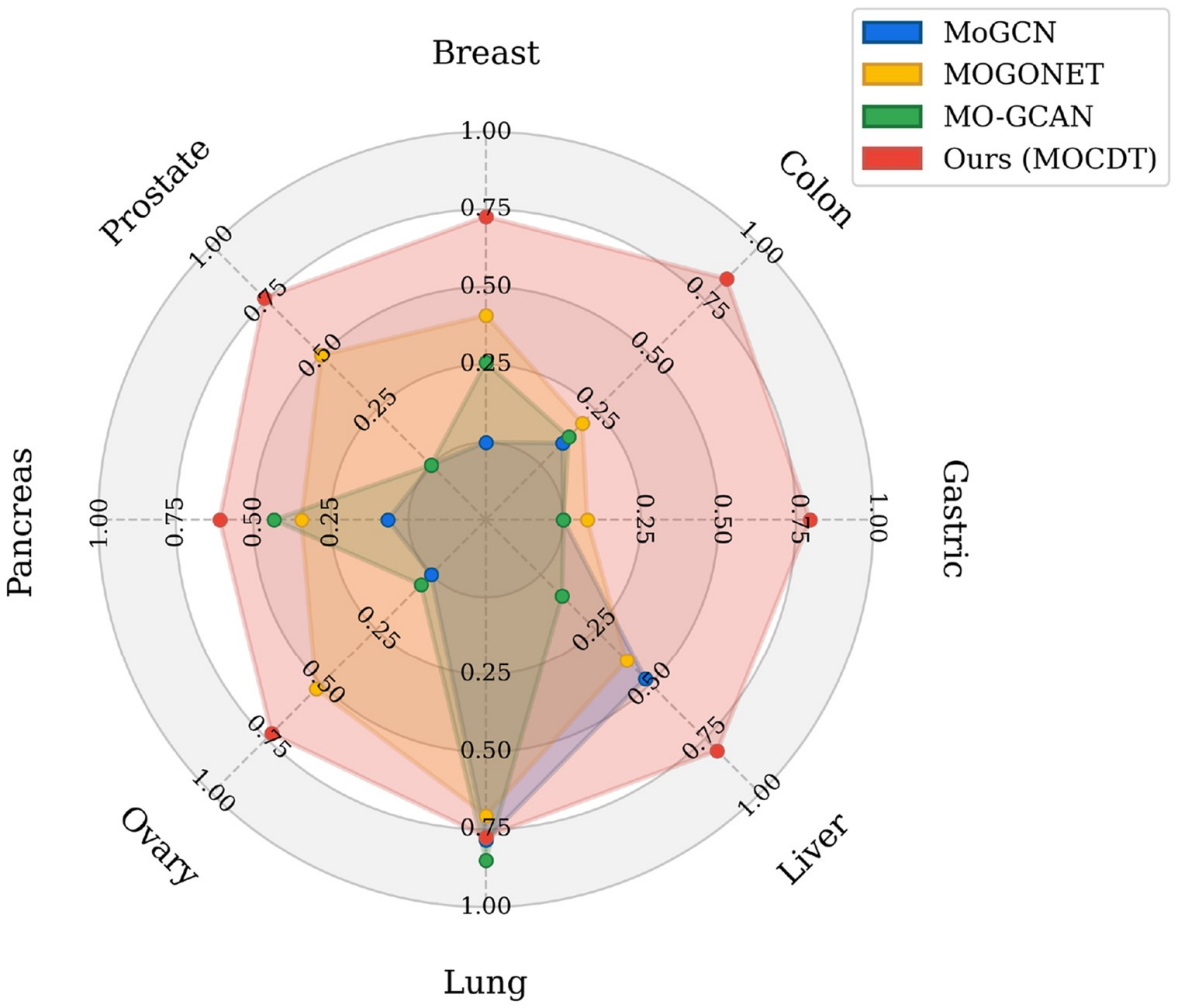
Per-cancer recall across eight cancer types for TOO, comparing MOCDT with benchmark approaches. Larger values indicate better recovery of true cancer cases for each cancer type.

We further quantified the across-class variance of each latent feature. To assess cancer-type-selective heterogeneity, we ranked latent features by their cross-cancer variance in mean feature importance (Fig. 3). Features with higher variance (upper rows) exhibit greater cancer-type specificity, whereas lower-variance features (lower rows) reflect more uniform, pan-cancer importance. This indicates that the learned latent space captures both cancer-type-specific and shared components. Additional visualization of the top-variance features is provided in Fig. 4, available as supplementary data at *Bioinformatics* online.

### 3.3 Comparison with benchmark studies

We compared MOCDT with MoGCN, MOGONET, and MO-GCAN under a clinically aligned CD→TOO pipeline (Table 1). For CD, decision thresholds were calibrated via stratified four-fold cross-validation to achieve target specificities. MOCDT performed best at 0.99 specificity, whereas baselines performed best at 0.95. At higher specificity levels, some baselines became overly conservative, yielding no CD-positive cases for downstream TOO evaluation. We therefore additionally compared all models at matched specificity levels (0.95 and 0.99), with sensitivity reported in Table 6, available as supplementary data at *Bioinformatics* online.

**Figure 5:**
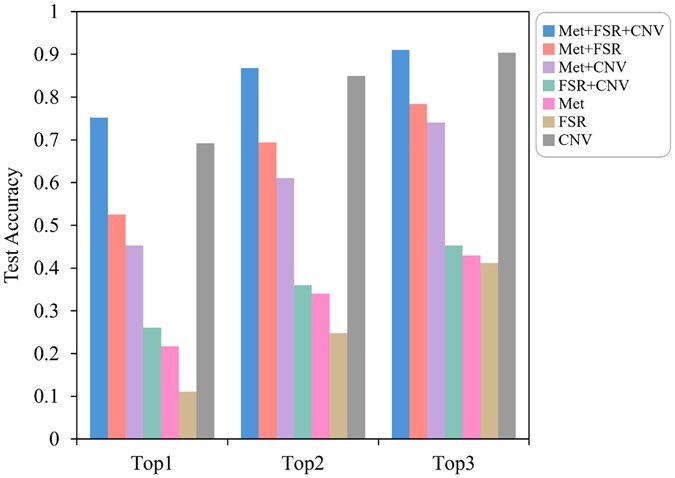
Top-K TOO classification accuracy of MOCDT across different modality combinations.

MOCDT also outperformed all baselines on TOO, achieving 0.7645 accuracy on ground-truth cancer samples and Top1/Top2 accuracies of 0.7520/0.8679 on the cancer-positive subset. Consistent with these results, Fig. 4 and Fig.5, available as supplementary data at *Bioinformatics* online, present radar plots of per-cancer recall and precision, showing that MOCDT outperforms baselines across most cancer types.

### 3.4 Comparison on ablation experiments

We conducted a progressive ablation study to quantify the contribution of each component in MOCDT. Starting from the full model, which combines a supervised multi-modal autoencoder, latent space SNF, and a residual GCN classifier, we evaluated four cumulative ablations: (1) replacing ResGCN with a vanilla GCN, (2) constructing SNF directly from raw features, (3) removing contrastive learning, and (4) removing adversarial learning (Table 2). To assess the contribution of each component, we also performed an ablation study by removing each major component from the final model while keeping the others unchanged (Table 7, available as supplementary data at *Bioinformatics* online), and further compared MOCDT with a Multi-Omics Factor Analysis-based integration baseline (Table 8, available as supplementary data at *Bioinformatics* online).

CD performance remained robust across ablations, suggesting that binary prediction relies on global cfDNA patterns preserved in the latent representation; sensitivity increased as components were removed, allowing more samples to enter the TOO stage. In contrast, TOO accuracy declined from 0.7520 to 0.7075 on predicted cancer samples. The largest performance drops were observed when removing contrastive or adversarial learning, highlighting their complementary roles: adversarial learning reduces modality gaps, while contrastive learning enhances class-discriminative structure in the latent space. Replacing latent SNF with raw SNF also reduced performance, indicating that latent similarity networks capture more informative relationships than those from high-dimensional inputs. This suggests that the autoencoder not only compresses features but also enhances biologically relevant structure for graph construction. Removing ResGCN or adversarial learning reduced TOO performance, while removing latent SNF or contrastive learning slightly increased CD accuracy but lowered specificity (Table 7, available as supplementary data at *Bioinformatics* online). Because high specificity is essential for cancer screening, CD accuracy alone is not sufficient to evaluate each component. Overall, MOCDT’s components provide complementary benefits, improving TOO while preserving robust CD performance.

### 3.5 Evaluation of modality integration

The comparative analysis of modality combinations in Table 9, available as supplementary data at *Bioinformatics* online, shows that integrating all three modalities—DNA methylation, FSR, and CNV—yields the best performance for both CD and TOO. In CD, the full multi-modal configuration achieves the highest accuracy (0.9609) with balanced sensitivity (0.9622) and specificity (0.9574). By contrast, single-modality models show a bias toward either sensitivity or specificity, indicating limited robustness.

For TOO classification on cancer-positive samples, MOCDT consistently attains the highest Top-K accuracy across all modality settings, reaching 0.9106 at Top3 (Fig. 5). CNV alone showed strong TOO performance, especially in Top2 and Top3 accuracy, but adding one more modality did not consistently improve performance. In contrast, integrating all three modalities yielded the most robust overall CD and TOO performance, indicating that full multi-modal integration provides the best results.

## 4 Discussion

Blood-based cfDNA MCED is required to operate under an extremely low false-positive rate in screening populations while providing reliable TOO classification to guide downstream diagnostic workup. Motivated by this setting, we propose MOCDT, a cfDNA multi-omics framework that integrates robust multi-modal representation learning with patient–patient relational modeling. The model effectively distinguishes tissue-specific signatures from universal malignancy markers by integrating structural instability from CNVs, regulatory plasticity from DNA methylation, and physical degradation patterns from fragmentomics. This biological synergy is crucial for overcoming the limitations of stochastic sampling in liquid biopsies and achieving the high specificity needed to minimize overdiagnosis in population screening.

This design is consistent with prior MCED studies emphasizing high-specificity detection followed by origin inference. Previous cfDNA-based multi-modal approaches and large-scale prospective evaluations of methylation-based MCED assays have reported >99% specificity together with accurate tissue- or cancer signal origin prediction among detected cases (Cohen *et al.* 2018, Marinac *et al.* 2025). Although direct numerical comparison is limited by differences in cohort design, cancer spectrum, stage distribution, and assay modality, these findings support the clinical relevance of coupling high-specificity CD with downstream origin prediction.

Methodologically, our results suggest that effective multi-modal cfDNA integration requires jointly regularizing cross-modality invariance and class-level separability. Adversarial alignment reduces modality-induced distribution shifts, while triplet objectives enhance healthy–cancer and tissue-specific boundaries, particularly improving TOO discrimination. Latent space similarity modeling with a lightweight residual GCN further leverages cohort structure and mitigates sensitivity to ultra-high-dimensional noise.

Despite these encouraging results, several limitations remain. Although CD thresholds were tuned for high specificity, operating points may vary across cohorts and require validation under distribution shift. Performance in truly asymptomatic screening populations also remains to be established, as ctDNA abundance is typically lower than in clinically diagnosed cohorts. Moreover, TCGA-based validation provides only indirect support for tissue-specific signals because of the cfDNA-to-tissue domain mismatch and should be complemented by external cfDNA cohorts or prospective studies. We are currently collecting clinical validation samples and plan to release the data for independent evaluation. Future work will focus on external validation, calibration under cohort shift, and error analysis across cancer types and stages.

## Supplementary Material

btag274_Supplementary_Data
