# Supplementary material for: MOCDT: multi-cancer detection and tissue-of-origin classification via cfDNA multi-modal integration
Source: Bioinformatics. 2026 Jul 7;42(Suppl 1):btag274. doi: 10.1093/bioinformatics/btag274 (PMC13340234; doi:10.1093/bioinformatics/btag274)
Supplement: btag274_Supplementary_Data [file btag274_supplementary_data.pdf]

Supplementary Material

1. Sample Processing and cfDNA Extraction

Whole blood samples were collected in EDTA tubes and processed within the recommended time window to minimize leukocyte lysis. Plasma was separated by two-step centrifugation, consisting of an initial spin at 1,500 × g for 15 min followed by a second high-speed centrifugation at 16,000 × g for 10 min to remove residual cellular debris. cfDNA was extracted from 2–4 mL of plasma using the Maxwell® RSC ccfDNA Plasma Kit (Promega, USA) according to the manufacturer's protocol. DNA concentration and fragment size distribution were assessed using the Cell-free DNA ScreenTape Assay on an Agilent 4200 TapeStation system. Samples showing evidence of high-molecular-weight genomic DNA contamination were excluded from downstream analysis. Genomic DNA from tumor tissue and matched healthy samples was extracted using the Maxwell® RSC Tissue DNA Kit (Promega, USA) for reference analyses.

2. NGS Data Preprocessing

Raw sequencing reads in BCL format were converted to FASTQ using Illumina bcl2fastq (v2.20.0.422). Quality control and adapter trimming were performed using fastp (v0.23.1). The processed reads were aligned to the GRCh38 (hg38) human reference genome using bitmapperBS (v1.0.2.1). Subsequent BAM file manipulations, including sorting and indexing, were conducted using SAMtools (v1.14). PCR duplicates were identified and removed using the MarkDuplicates function of GATK (v4.2.3.0). Final quality metrics required a mapping rate $> 80\%$, a duplication rate $< 25\%$, and a methylation conversion efficiency $> 99\%$.

3. Data Preprocessing Details

We provide additional details on preprocessing steps complementing the summary in Section 2.2. DNA methylation data originally comprised 476,229 CpG sites. Among these, 48,127 CpGs overlapped with 49,156 CpGs used during MethylGPT pretraining, while 350,149 CpGs contained no missing values across all samples. We constructed a methylation feature set consisting of 363,987 CpGs obtained by taking the union of (i) CpGs overlapping with those used during MethylGPT pretraining and (ii) CpGs in our dataset without missing values. These two groups shared 34,289 CpGs, resulting in a total of 363,987 CpGs after removing duplicated entries (48,127 + 350,149 - 34,289). Out-of-range imputed values were clipped to the biologically valid range [0,1]. For downstream tasks, supervised feature selection for the methylation modality was performed using the `limma` package Ritchie et al. (2015) with a binary cancer label (cancer vs. healthy) on the MethylGPT-imputed methylation matrix. CpG sites satisfying the significance threshold of $p < 0.05$ were retained, resulting in a final methylation feature set of 110,392 CpGs. For methylation data, MethylGPT-based imputation was applied only during the feature selection stage, while the original methylation values were used during model training with missing entries filled with zero. For the FSR modality, genomic regions with more than 50% missing values were removed, resulting in 25,461 retained regions out of 25,465. For the CNV modality, all 24,579 regions were retained, as no missing values were observed. For FSR, missing values were also replaced with zero, and all feature matrices were formatted into unified tabular inputs for downstream modeling.

4. Additional Experimental Results

Table S1. Summary of the study cohort across cancer type, sample type, source institution, number of samples, and IRB approval number.

Cancer Type	Sample Type	Source	Number of Samples	IRB No.
Healthy	blood	SNUH	140	H-2201-128-1294
	blood	HMF	142	HNR2022-02
	plasma	SNUH	83	H-1805-049-944
Colon	plasma	SNUH	131	H-1708-031-875
	tumor tissue	SNUH	106	H-1708-031-875
	adjacent normal tissue	SNUH	20	H-1708-031-875
Liver	plasma	AJUH	62	AJIRB-BMR-EXP-21-525
	plasma	SNUH-HUB	51	H-2201-128-1294
	tumor tissue	AJUH	30	AJIRB-BMR-EXP-21-525
	tumor tissue	SNUH-HUB	20	H-2201-128-1294
	adjacent normal tissue	AJUH	26	AJIRB-BMR-EXP-21-525
Lung	plasma	SMC	125	2022-10-034
	plasma	SNUH	14	1907-090-1048
	plasma	SNUH-HUB	100	H-2201-128-1294
	tumor tissue	SNUH-HUB	16	H-2201-128-1294
	tumor tissue	SNUH	15	1907-090-1048
	adjacent normal tissue	SNUH	14	1907-090-1048
Prostate	plasma	EUMC	135	SEUMC 2022-08-047-002
	plasma	SNUH-HUB	8	H-2201-128-1294
	tumor tissue	SNUH-HUB	30	H-2201-128-1294
Breast	plasma	SNUH-HUB	74	H-2201-128-1294
	tumor tissue	SNUH-HUB	50	H-2201-128-1294
Ovarian	plasma	SNUH-HUB	87	H-2201-128-1294
	tumor tissue	SNUH-HUB	34	H-2201-128-1294
Gastric	plasma	NCC	100	NCC2022-0252
	tumor tissue	NCC	32	NCC2022-0252
Pancreatic	plasma	SNUH-HUB	13	H-2201-128-1294
	plasma	SMC	87	2016-07-054
	tumor tissue	SMC	39	2016-07-054

Table S2. Number of samples in the train and test sets for each class, including healthy and eight cancer types.

	Healthy	Breast	Colon	Gastric	Liver	Lung	Ovary	Pancreas	Prostate	Total
Train set	193	49	62	55	83	120	48	71	51	732
Test set	188	46	59	45	80	131	46	47	49	691

Table S3. Sensitivity analysis of MethylGPT-assisted feature selection. "Without MethylGPT-assisted feature selection" denotes the setting in which methylation feature selection was performed after zero imputation instead of MethylGPT-based imputation. In both settings, model training used the original methylation signals, with missing selected values filled with zero.

Setting	Cancer Detection			TOO (GT Cancer)	TOO (Pred Cancer)
	Accuracy	Specificity	Sensitivity	Accuracy	Accuracy
MOCDT (Proposed)	**0.9609**	**0.9574**	0.9622	**0.7645**	**0.7520**
Without MethylGPT-assisted feature selection	0.9595	0.9255	**0.9722**	0.7239	0.7038

Table S4. Precision and recall of MOCDT stratified by gender.

	Female		Male	
Cancer type	Precision	Recall	Precision	Recall
Breast	**0.9143**	0.7273	–	–
Colon	0.8077	**0.8750**	0.8286	0.8286
Gastric	0.6667	0.8235	0.8400	0.7778
Liver	0.6667	0.6316	**0.8929**	**0.8621**
Lung	0.6429	0.7105	0.8313	0.8023
Ovary	0.6154	0.7273	–	–
Pancreas	0.7143	0.5000	0.6207	0.6923
Prostate	–	–	0.7778	0.7609

Table S5. Precision and recall of MOCDT stratified by cancer stage. Early stage includes stages 1-2, and late stage includes stages 3-4.

	Early stage		Late stage	
Cancer type	Precision	Recall	Precision	Recall
Breast	0.8846	0.9583	0.7500	0.4500
Colon	0.8333	**0.9615**	0.8333	0.7576
Gastric	**0.9667**	0.8056	0.3750	0.7500
Liver	0.9455	0.8000	0.5882	**0.8333**
Lung	0.7143	0.7692	**0.8049**	0.7765
Ovary	0.5294	0.7500	0.6053	0.7188
Pancreas	0.5789	0.6471	0.7083	0.5862
Prostate	0.8125	0.8125	0.6429	0.6429

Table S6. Performance comparison of CD and TOO classification with benchmark models evaluated at target specificities of 0.95 and 0.99 for cancer detection. For the TOO evaluation, Top-K accuracy measures whether the ground-truth cancer type is contained within the top-K predicted classes ranked by posterior probability, reflecting clinically relevant diagnosis scenarios.

	Target	Cancer Detection			TOO (GT Cancer)	TOO (Predicted Cancer)			# of Predicted
	Specificity	Acc	Spec	Sens	Acc	Top1 Acc	Top2 Acc	Top3 Acc	Cancer
MoGCN	0.99	0.4226	0.9947	0.2087	0.2803	0.3333	0.4952	0.6857	106
	0.95	0.6657	0.9681	0.5527	0.2803	0.3237	0.5396	0.6691	284
MOGONET	0.99	0.2720	**1.0000**	0.0000	0.3718	-	-	-	0
	0.95	0.5790	0.9260	0.4490	0.3976	0.4167	0.5625	0.6667	240
MO-GCAN	0.99	0.2721	**1.0000**	0.0000	0.3042	-	-	-	0
	0.95	0.6151	0.6915	0.5865	0.3042	0.2720	0.4164	0.4901	353
MOCDT (Proposed)	0.99	**0.9609**	0.9574	0.9622	**0.7645**	**0.7520**	**0.8679**	**0.9106**	492
	0.95	0.9551	0.8564	**0.9920**	0.7615	0.7224	0.8365	0.8802	526

Table S7. Performance comparison of ablation experiments. ((-)ResGCN: No residual layers for GCN, (-)Latent SNF: Use raw input data to construct SNF, (-)Cont: No contrastive learning, (-)Adv: No adversarial learning).

	Cancer Detection			TOO (GT Cancer)	TOO (Predicted Cancer)
	Accuracy	Specificity	Sensitivity	Accuracy	Accuracy
MOCDT (Proposed)	0.9609	**0.9574**	0.9622	0.7645	0.7520
(-)ResGCN	0.9682	**0.9574**	0.9722	0.7587	0.7465
(-)Latent SNF	0.9638	0.9415	0.9722	**0.7730**	**0.7560**
(-)Cont	**0.9711**	0.9415	0.9821	0.7713	0.7545
(-)Adv	0.9682	0.9202	**0.9861**	0.7500	0.7280

Table S8. Comparison with an alternative multimodal integration baseline, Multi-Omics Factor Analysis (MOFA). In the MOFA-based variant, multimodal latent factors integrated by MOFA were used and evaluated with the same downstream graph classifier.

	Cancer Detection			TOO (GT Cancer)	TOO (Predicted Cancer)
	Accuracy	Specificity	Sensitivity	Accuracy	Accuracy
MOCDT (Proposed)	**0.9609**	**0.9574**	**0.9622**	**0.7645**	**0.7520**
MOFA	0.8683	0.8191	0.8867	0.2063	0.1917

Table S9. Performance comparison of cancer detection and tissue-of-origin (TOO) classification across different modality combinations. TOO performance was assessed on both ground truth samples and samples predicted as cancer by the detection model. The first row corresponds to the proposed MOCDT framework.For modality-combination experiments, the tri-modal model used a latent fusion ratio of 2:4:4, whereas bi-modal models used equal weighting (5:5). (Met: DNA methylation)

	Cancer Detection			TOO (GT Cancer)	TOO (Predicted Cancer)		
Modality	Accuracy	Specificity	Sensitivity	Accuracy	Top1 Acc	Top2 Acc	Top3 Acc
Met + FSR + CNV	**0.9609**	0.9574	0.9622	**0.7645**	**0.7520**	**0.8679**	**0.9106**
Met + FSR	0.7164	0.9309	0.6362	0.5469	0.5255	0.6937	0.7838
Met + CNV	0.7077	0.9202	0.6282	0.4747	0.4532	0.6103	0.7402
FSR + CNV	0.7265	0.0319	**0.9861**	0.3569	0.2611	0.3599	0.4528
Met	0.7742	0.2340	0.9761	0.2811	0.2173	0.3402	0.4299
FSR	0.7366	0.6968	0.7515	0.1270	0.1103	0.2483	0.4115
CNV	0.9580	**0.9840**	0.9483	0.6960	0.6917	0.8500	0.9042

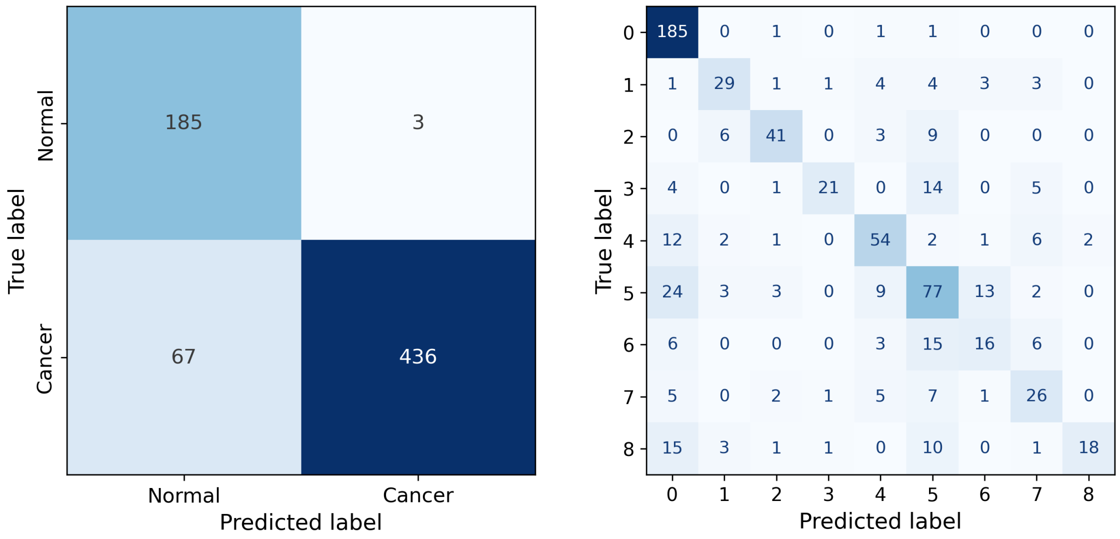

Fig. S1: Confusion matrices of autoencoder classifier on the test set. (Left) Binary cancer prediction (healthy vs cancer). (Right) TOO classification result includes healthy (label: 0). The autoencoder classifier mainly functions as an initial representation learning and coarse prediction module, while the relatively higher number of false negatives observed in the autoencoder stage is reduced after the GCN refinement.

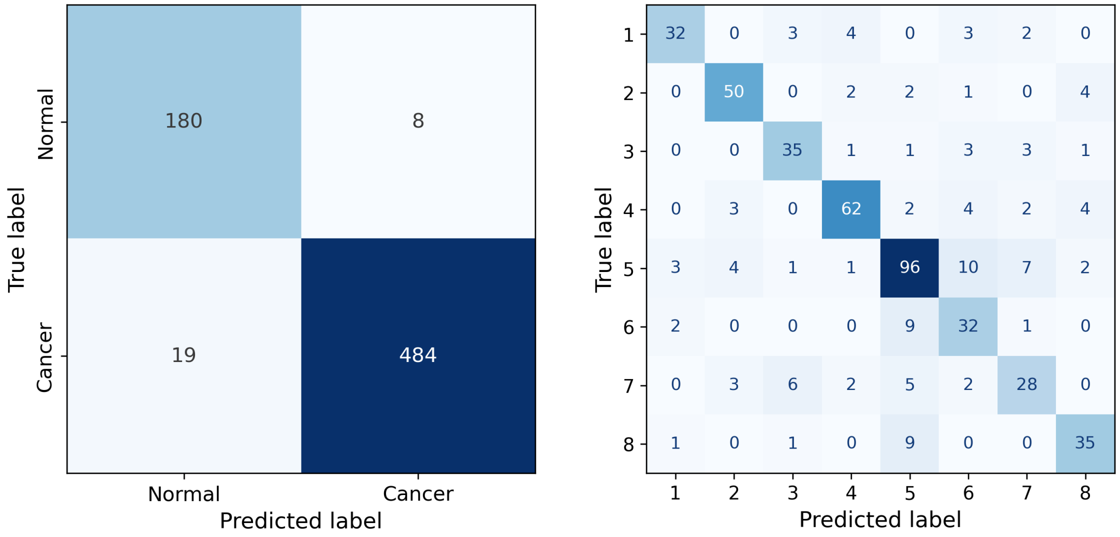

Fig. S2: Confusion matrices of MOCDT on the test set. (Left) Binary cancer prediction (healthy vs cancer). (Right) TOO classification evaluated on the subset of samples predicted as cancer.

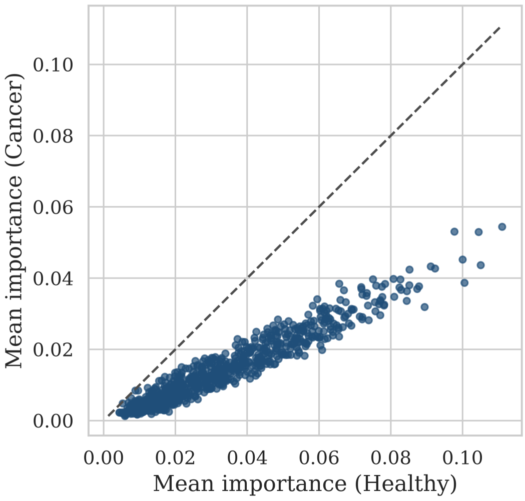

Fig. S3: Latent feature importance for healthy vs cancer samples. Each dot corresponds to one latent feature, with axes indicating mean gradient-based importance in healthy and cancer groups.

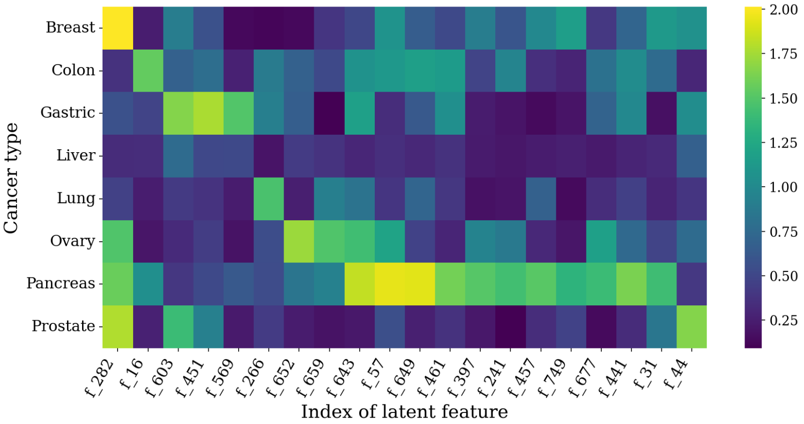

Fig. S4: Heatmap of mean importance for top 20 variance latent features across cancer types. Latent features are grouped according to their dominant cancer type. The visualization reveals block-wise activation patterns, where subsets of features are selectively important for specific cancer types, indicating structured, cancer-specific latent representations. $f\_\#$ denotes the index of each latent feature.

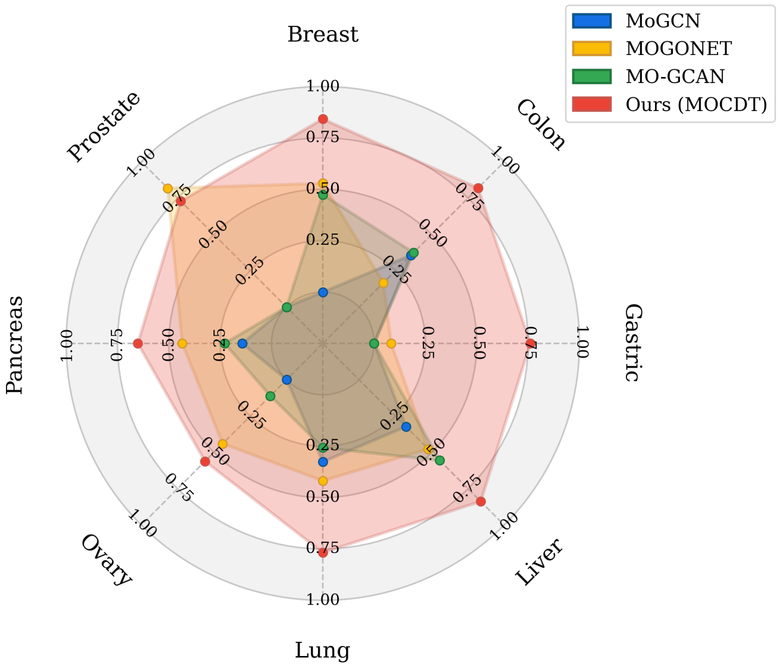

Fig. S5: Per-cancer precision across eight cancer types for tissue-of-origin (TOO), comparing MOCDT with MoGCN, MOGONET, and MO-GCAN. Larger values indicate better recovery of true cancer cases for each cancer type.
